# Evidence for side effects of cannabidiol (CBD) products and their non-conformity on the European food market – response to the European Industrial Hemp Association

**DOI:** 10.12688/f1000research.26045.1

**Published:** 2020-08-26

**Authors:** Dirk W. Lachenmeier, Stephan G. Walch

**Affiliations:** 1 Chemisches und Veterinäruntersuchungsamt (CVUA) Karlsruhe, Karlsruhe, 76187, Germany

**Keywords:** Tetrahydrocannabinol, cannabidiol, Cannabis sativa, hemp, food supplements, risk assessment, drug effects

## Abstract

An interesting and valuable discussion has arisen from our recent article (Lachenmeier et al., 2020) and we are pleased to have the opportunity to expand on the various points we made. Equally important, we wish to correct several important misunderstandings that were made by Kruse and Beitzke (2020) on behalf of the European Industrial Hemp Association (EIHA) that possibly contributed to their concerns about the validity of our data, toxicological assessment and conclusions regarding regulatory status of cannabidiol (CBD) products. First and foremost, our study did only assess the risk of psychotropic Δ ^9^-tetrahydrocannabinol (THC) without inclusion of non-psychotropic Δ ^9^-tetrahydrocannabinolic acid (THCA). Secondly, as this article will discuss in more detail, there is ample evidence for side effects of CBD products, not only in paediatric patients, but also in adult users of over-the-counter CBD products (including inadvertent “high” effects). Thirdly, the exposure and risk assessment was conducted using up-to-date guidelines according to the European Food Safety Authority (EFSA) and the German Federal Institute for Risk Assessment (BfR). And finally, the current legal situation in the European Union, without approval of any hemp extract-containing product according to the Novel Food regulation, actually allows blanket statements that all such products are illegal on the market, and this indeed would imply a general ban on the use and marketing of such products as food or food ingredients until such an approval has been granted. We hope that this reassures the F1000Research readership regarding the validity of our results and conclusions. We are pleased, though, that the EIHA has acknowledged the fact that there are non-compliant CBD products available, but according to our data these are a substantial fraction of the market.

## Introduction

We actually agree with a main premise of the European Industrial Hemp Association (EIHA)’s comments; namely, that legal compliance and safety for both producers and consumers of cannabidiol (CBD) products must be ensured ^1^. If this can be achieved by their suggestion of a mandatory industry self-regulated approach ^1^ rather than by our suggestion of strict regulations ^2, 3^ is another question. In light of the experience with industry self-regulations in other fields, this suggestion remains highly doubtful ^4, 5^; it especially appears not well-thought-out how a self-regulation may be mandatory and how this demand can be enforced. Otherwise, we had previously suggested the necessity for a common regulatory approach regarding hemp food products on a European level, such as enforceable maximum levels for Δ ^9^-tetrahydrocannabinol (Δ ^9^-THC) ^6^. To even increase the legal void by the assessment of hemp extract-based food products as non-foods within the international and national narcotic regulations ^7^ is clearly not helpful.

Apart from these issues, which are political rather than scientific, the main finding presented in our study is that the levels of psychoactive Δ ^9^-THC in many CBD products on the market exceed acceptable thresholds of toxicity. Furthermore, hemp extract-based CBD products were assessed as unapproved novel foods. For both reasons, the marketing of such products is illegal according to European Union (EU) food laws ^2^ (if we assume that the products are foods and not narcotics). The disputation provided by EIHA to refute our assessment is based on claims rather than facts and we therefore take this chance to corroborate our assessment by further data published since the last revision of our paper in January 2020.

Let us now consider each criticism of the EIHA ^1^ in detail:

## Side effects of CBD products

The literature regarding side effects of CBD was considerably expanded since the writing of our article, so that besides the anecdotal reports and paediatric studies already mentioned, several case reports ^8– 10^, a survey ^9^, a meta-analysis of clinical trials ^11^ and a post marketing safety assessment of a full spectrum hemp extract ^12^ became available. Specifically the survey ^9^ reports observations of side effects including “feeling high”, an effect likely to be explained by Δ ^9^-THC contamination rather than by CBD. Similarly, effects of “a little high”, brief periods of mild intoxication, were described following ingestion of several brands of CBD products in Virginia, USA ^10^. The post marketing safety assessment showed gastrointestinal effects as most commonly reported side effect ^12^. We have included this information into a new version (version 3; v3 ^13^) of our article ^2^ to strengthen our arguments. Besides the mentioned human evidence, experimental research *in vivo* and *in vitro* resulted in concerns about hepatotoxicity ^14^, teratogenicity ^15^, and gut inflammation ^16^.

The arguments of EIHA in refuting any side effects of CBD products are not convincing not only from a pharmacological standpoint, but it is also rather unscientific to refute side effects on the basis that the responsible authority in the UK has not been made aware of any safety incident till now ^1^. First, due to the very short time of public use of CBD, only acute toxic events would currently appear, while chronic toxic events, such as liver toxicity, may take years to develop. Second, there is currently no system of nutrivigilance implemented in the UK or most other EU member states, so that there is no formal registration of such cases.

## “THC” definition and estimation of daily dose of products

Unfortunately, a misunderstanding regarding our definition of Δ ^9^-THC has apparently occurred at EIHA. From the methods section and our definition of the abbreviation of THC as Δ ^9^-tetrahydrocannabinol, it should be clear that we only include the psychotropic Δ ^9^-THC and not the non-psychotropic Δ ^9^-tetrahydrocannabinolic acid (THCA) in our risk assessment. In deviation of the suggested practice to implement the German guidance values for total THC (i.e. the sum of Δ ^9^-THC and THCA) ^17^, we only have compared the psychotropic Δ ^9^-THC with the guidance values. This practice is clearly in favour of the food business operator (FBO) because – as the EIHA correctly states – a major part of total THC may be comprised by THCA ^1^. We have updated our article to clarify this issue on several instances and to avoid future misunderstandings ^13^.

Regarding the exposure assessment, we confirm to have conducted an estimation of the daily intake in those instances, where a maximum recommended daily dose was missing in the labelling. This is in accordance with the requirements of Art. 14 of Regulation (EC) No. 178/2002 laying down the general principles and requirements of food law ^18^, which specifies that the information provided to the consumer must be considered in determining whether any food is unsafe. As we will elaborate further in the following, our exposure estimations are both practical and realistic from the standpoint of consumer health protection.

### Tea products (hemp flowers or leaves)

The authors consider 8 g of tea product consumption per day as an absolutely common and realistic scenario, e.g. comparable to consuming 1 teapot (for example 8 g infused in 1 L of water). The German Federal Institute for Risk Assessment (BfR) even suggests a slightly higher amount of 2 g / 200 mL infusion (10 g/L) and suggests that the actual acute consumption quantities for herbal tea as an analogon for hemp tea are in the order of 1.3 litres (P95) ^19^. Regarding the question of carry-over of THC into the infusion, the BfR has recently reviewed the evidence including the study cited by EIHA ^20^ and another study by our group ^21^. The BfR concluded: “The BfR is of the opinion that the assumption of 100% carryover is justified, as experimental data on the carryover point to high fluctuations” ^19^. Therefore, we currently fail to see the evidence to change our exposure assessment for tea. Perhaps the EIHA can submit their unpublished test reports (see footnote 10 in Kruse & Beitzke ^1^) to the BfR for re-evaluation of their opinion, or even preferably make them publicly available in the form of a dataset for their article ^1^.

Finally, the allegation of the EIHA that we “ignored or overruled” the recommended daily dose on the label or the brewing instruction of the FBO ^1^ is untrue, as we certainly used this information when available (see dataset ^22^).

### Syrup with hemp flower extract

According to the labelling, the syrup is intended to be used to prepare a beverage in 1:10 dilution with water. According to the BfR recommendation for tea, we assumed the use of 130 ml syrup to prepare 1.3 L of final beverage. The consumption of this amount of alcohol-free beverage is certainly not excessive nor is our assumption arbitrary or results-oriented. We would like to explicitly reject this unscientific and unfounded accusation of the EIHA ^1^ that the CVUA Karlsruhe or its employees’ work is results-oriented, in the sense that we exaggerate the risk of hemp products aiming to prohibit them from the market. On the contrary, the CVUA Karlsruhe works in its expert activities completely independent from any interests and our highest goal is the protection of the consumer from health damage.

### Cannabis shot

There appears to be a misunderstanding about what is a “shot”. A shot is a form of concentrated beverage to be consumed as such and it is not a food supplement. The flask contains a single whole portion of the “shot” intended by the manufacturer to be consumed at once (e.g. compare “shots” of energy drinks). The “shot” is therefore clearly a “ready-to-eat” product, which is covered by the German guidance values.

### CBD oil

Regarding the evaluation of so-called CBD oils, which are typically constituted of full spectrum hemp extracts mixed into edible oils to achieve CBD concentrations in the range 5–15% being sold as food supplements, the allegation of EIHA that we dismiss the many cases in which the dosage or recommended daily intake was provided correctly by the respective manufacturers ^1^, must be clearly rebutted. As can be seen in our dataset ^22^, as well as in Table 2 of our article ^2^, we have consistently and unambiguously used the labelled dosage of the manufacturers for the comparison with the toxicological thresholds. Naturally, for the products where no dosage had been labelled, exposure had to be estimated similar to the estimation for the beverages discussed above.

The product under specific scrutiny of EIHA is a special case as it was only labelled as “CBD oil” with no labelling suggesting it to be a “food supplement”. Therefore, the discussion regarding what consumers might expect from food supplements is not helpful. We believe that consumption of 10 ml (about 1 tablespoon) of an oil that is not labelled as “supplement” or with any other warning labels, is not an exaggerated or unrealistic scenario in all objectivity. It must also be considered that the THC content in this product was so high, that the consumption of 1/10 of the amount (i.e. 1 mL) would also exceed the lowest observed adverse effect level (LOAEL) and therefore lead to the same outcome. Nonetheless, we have clarified footnote 2 in Table 2 in the v3 of our article ^13^ expanding the explanation of our exposure assessment in this case. It must be noted, however, that even if we would exclude this clearly exceptional and outlying product from our sample collective, all results and conclusions of our article are still valid. We are also surprised that the EIHA takes offence in our activities and responsibilities as part of governmental food control in Germany, while the problem clearly lies with FBOs that mislabel and misrepresent their products. Furthermore, for each of the products in Table 2 of our article ^2^, detailed expert opinions were produced for the responsible food control authorities that had initially submitted the samples to our institute. In some cases, our expert opinions have become part of court proceedings and the courts have confirmed the risk assessment of the CVUA Karlsruhe, as well as the risk management measures of the authorities in all cases known to date ^23– 25^.

## Mitigation of THC effects by interaction with CBD?

The allegation of EIHA that we have dismissed the interaction between THC and CBD ^1^, in the sense that CBD would mitigate the effects of THC, can be clearly rebutted. First and foremost, the underlying risk assessment in our expert opinions is based on the opinion of the Panel on Contaminants in the Food Chain of the European Food Safety Authority (EFSA) ^26^, which has considered interaction effects. However, EFSA concluded the information is controversial and not consistently antagonistic ^26^. This is consistent with more recent research of Solowij *et al*. ^27^ that the effects of Δ ^9^-THC may even be enhanced by low-dose CBD (e.g., as found in food supplements) and may be particularly prominent in infrequent cannabis users. Positive findings regarding antagonistic effects (e.g. Pisanti *et al*. ^28^ cited by EIHA) were typically found for much higher dosing regimens, i.e. aiming to mitigate the adverse effects of THC in hashish and marihuana, while another study with smoked cannabis did not detect such an effect ^29^.

We strongly believe, in line with EFSA, that the current scientific evidence does not allow for considering cumulative effects in low dose CBD oils and hemp extracts. The applicability of the acute reference dose (ARfD) of 1 μg Δ ^9^-THC per kg body weight – without considering interactions by CBD – was recently re-confirmed by EFSA ^30^.

As the EIHA mentioned this argument, we have decided to include a short rationale into the v3 of our article ^13^ for reasons of completeness. Otherwise, our article is not a basic toxicological research article about the rationale for risk assessment but an applied research article, which has based the risk assessment on the guidelines of the responsible risk assessment authorities BfR ^19^ and EFSA ^26, 30^. Therefore, we would invite EIHA to correspond directly with these institutions, when they believe there is scientific evidence or new data that might change the available assessments. Currently, we see no such data. It should be noted that the EIHA has unsuccessfully tried lobbying the risk assessment bodies into providing more “reasonable” guidance values for THC (e.g., see Banas *et al*. ^31^), and we believe that a comment on our scientific article is not the right place to continue this effort.

## Illegality of all hemp products containing isolated CBD or hemp extracts

While the regulatory status is not part of our chemical and toxicological research, we thank the EIHA for pointing out this issue, as there is a potential misunderstanding of the lobbyist regarding the most up-to-date regulations and decisions of EU and national legislators as well as of the courts, which is evidenced by the outdated references cited by EIHA ^1^.

We also thank the EIHA ^1^ for the re-iteration of our conclusion that “basically all available CBD products based on hemp extract marketed as food or food supplement within the EU are therefore illegally sold”. We still stand by this conclusion.

It is certainly true that case-by-case decisions have to be conducted in official food control, and of course we have exactly done this for each product, which was submitted to our laboratory for evaluation. However, the situation of hemp-extracts is a particular one, because of its regulatory status as unapproved novel food. This status allows for such a blanket statement, that each single product that contains hemp extract as ingredient can be judged as illegally placed on the market. It should be noted that this assessment is independent of the amount of hemp extract or its concentration of CBD. Regarding the THC levels found, which are widely variable, a case by case decision has to be made in any case, which spans from unsuspicious levels below the German guidance levels up to exceedance of the LOAEL dose, which we judge as a serious risk in consideration of Art. 14 of Regulation (EC) No. 178/2002 ^18^.

The EIHA ^1^ is also correct in considering the EU Novel Food Catalogue, which leads to this “blanket” assessment of hemp extracts as being novel, as legally not binding and that it is only an indicator for court decisions. What the EIHA, however, fails to mention is the fact that there are a number of court decisions that have actually endorsed the suggestions of the novel food catalogue and have confirmed the actions of the authorities in prohibiting the placing of the respective CBD product on the market ^24, 25, 32– 38^. To our knowledge, there currently is no court ruling, that might have endorsed the EIHA opinion.

Furthermore, the court rulings have also disproved the claims of the EIHA about the burden of proof for determining the novelty of a food. The opinion of EIHA ^1^ in this regard is based on outdated, incomplete evidence. In their decision about the marketability of a CBD product, the administrative court of the German Federal State Baden-Württemberg ruled that the food business operator has the burden of proof ^25^. This is in accordance with Article 4(1) of Regulation (EU) No 2015/2283, which states that the food business operator shall verify that foods which he or she has placed on the market in the EU, fall within the scope of this Regulation or not ^39^. Also outside the CBD field, the burden of proof has been imposed on the FBOs in several court rulings confirming Art. 4(1) of Regulation (EU) No 2015/2283 (see review of court rulings in Meyer *et al*. ^40^). For a more detailed assessment of CBD court rulings see our recent review ^41^.

Finally, we cannot follow the arguments of the EIHA ^1^ that European Court of Justice decisions regarding pharmacological effects might be relevant or that the novelty of a product is connected with an associated abstract health risk. The novelty of a product purely depends on the fact that it was not used for human consumption to a significant degree within the EU before 15 May 1997 ^39^. The novelty does not depend on potential pharmacological effects or health risks of the product.

The German Federal Office of Consumer Protection and Food Safety (BVL) recently published a statement that the classification of food containing CBD in the press release of EIHA of March 3, 2020, is not correct ^42^. The BVL states that for extracts of *Cannabis sativa* L. and derived products containing cannabinoids (e.g. CBD) a significant history of consumption in the EU has still not been demonstrated by the economic operators, nor by the EIHA or any other association ^42^. For this reason, they are still considered EU-wide as novel foods ^42^.

In conclusion, we believe that the responsible authority can currently make conclusions on the non-marketability of CBD products based on a lack of novel food approval, and additionally based on the lack of safety when THC thresholds are exceeded. We must stress here that the responsible local authority’s tasks clearly include the enforcement of the Novel Food Regulation ^39^ as well of the food safety rules ^18^. This is practiced all over Europe and evidenced by the numerous alerts found in the EU’s Rapid Alert System for Food and Feed (RASFF) ^43^.

## Judgement about food producers of CBD products

Since the publication of our article ^2^, a number of studies have confirmed our analytical results. Food control authorities in Europe have reported various offences of FBOs selling CBD products against the European food law. More than 150 notifications regarding CBD as unauthorised novel food ingredient and/or unauthorised THC in CBD products were shared in the RASFF. In Belgium, about half of 213 products seized from CBD shops exceeded a threshold of 0.2% THC+THC-A and large discrepancies were observed between labelled and measured CBD concentration ^44^. The Food Safety Authority of Ireland (FSAI) reported that from 38 tested CBD products, 37% exceeded the safe limit of THC dosage set by EFSA (1 µg/kg body weight/day), 34% were classified as novel food lacking approval, 36% were food supplements lacking the necessary notification of the competent authority, 92% were tested to contain differences between analytical and declared CBD content of more than 10%, and finally 50% contained misleading claims such as unauthorised health claims or medicinal claims ^45^. An analysis of over-the-counter CBD products from the UK found that only 38% of 29 products were within 10% of advertised CBD content and 55% had measurable levels of THC or cannabinol ^46^. Similarly, only 3 out of 25 cannabidiol products from the State of Mississippi (USA) were within 20% of label claim, and 3 exceeded 0.3% THC ^47^. Similar studies from Italy ^48^, the Netherlands ^49^, and the USA ^50^ are available.

In consideration of these consistent reports worldwide, we actually cannot find a better wording than our original statement: “In our opinion the systematically high Δ ^9^-THC content of CBD products is clearly a “scandal” on the food market. Obviously, the manufacturers have – deliberately or in complete ignorance of the legal situation – placed unsafe and unapproved products on the market and thus exposed the consumer to an actually avoidable risk.”

We fully stand by this conclusion and have even expanded our judgement of the CBD industry in a recent editorial, which concluded that the illegal market of CBD products may provide a strong rationale for the necessity of a paradigm shift towards pre-marketing approval in regulating food supplements ^3^.

The following arguments of EIHA ^1^, starting with obsolete letters of the EU commission (written at a time when hemp extracts were not available on the market, highlighting their irrelevance to the current situation) and some disconnected information about novel food status, without providing any evidence at all besides unsubstantiated claims, cannot plausibly refute our conclusions. Instead, we have provided ample evidence – based on EFSA criteria ^26^ – that a substantial number of CBD products on the market is not safe (69% of samples above ARfD of EFSA) and all samples (100%) were judged on a case-by-case basis as unapproved novel foods. Additionally, all samples (100%) were non-compliant with mandatory labelling rules and/or used unapproved health claims ^2^. We feel that this is ample proof for our statement above, which is based on facts.

The CVUA Karlsruhe as part of the food control system in the EU also clearly wishes to reject the allegation of EIHA ^1^ that the institute conducts “discrimination”, “undifferentiated action” and “arbitrariness”. We have assessed all products sent to our institute for evaluation in a transparent and consistent fashion (the criteria for evaluation were published in 2019 ^51^), conducted our toxicological and regulatory assessment on a case-by-case basis ^2^, and even allowed public scrutiny by publishing our full dataset ^22^.

Regarding the concerns of EIHA to defend the reputable hemp industry against “free riders”, “black sheep” or “cowboys”, we can ensure them that food control includes this segment of the market as well, e.g. by conducting sampling of online stores. Otherwise, the EIHA has the possibility to take their own steps against such practices on the basis of the national laws against unfair commercial practices (e.g. in Germany “Gesetz gegen den unlauteren Wettbewerb (UWG)”).

The closing remarks of EIHA ^1^ in this section appear ill-considered. First, it is commendable that EIHA wants to ensure compliance with the law and consumer safety. But how can this solely be achieved by an industry standard? And how can an industry standard be made mandatory for all FBOs? Perhaps on a voluntary basis for the members of EIHA, but clearly not for the whole industry, and not for the “free riders”, “black sheep” or “cowboys”. As stated before, we would certainly agree with an improved legal basis for hemp food products similar to other vertical regulations in the food sector, such as the EU spirit drinks regulation. However, we fail to see how this can be achieved as an industry standard.

Regarding the lack of communication between EIHA and public authorities, we recall a technical discussion at our institute at the end of 2018 and are also aware that the EIHA was invited to present their evidence at the “Working Group Novel Food” in Brussels ^52^.

Finally, we congratulate the EIHA for the decision to facilitate novel food applications by conducting extensive toxicology studies.

## Judgement of the hemp industry in the food sector

The quote "Currently CBD users must be aware that they may be ‘participating in one of the largest uncontrolled clinical trials in history’” of Pál Pacher included in a Newsweek article ^53^ is in our opinion very fitting to the reality of the market. First, Pál Pacher is clearly an authority regarding cannabis research (e.g., Refs. ^54– 57^). Second, the comment is regarding CBD and not regarding THC, and we currently cannot see a substantial difference between CBD content of food or nutritional supplements on the markets in the USA and Europe. Along with the lack of labelling detected in our study and the suggestions of many manufacturers to “gradually increase the dosage”, pharmacologically active CBD dosages similar to prescription medications may be easily reached by commercial over-the-counter CBD products on the market in Europe. As noted above, no nutrivigilance is typically conducted and no safety assessment has been conducted for the products, because the manufacturers put them on the market before achieving novel food approval.

## Proposal of a legal ban on hemp extracts

We wonder why our statement “For cannabis-derived products, such as CBD, the problem is aggravated by conflicting regulations in the narcotic, medicinal, and food law areas. For example, hemp extract based products of similar composition could be treated as illegal narcotics, prescription-based medicinal products, or novel foods” is criticized by the EIHA ^1^, when they actually provide supporting evidence with their examples of melatonin or garlic that certain substances could fall into either legal realm depending on labelling sometimes even when the concentration is similar (e.g., also compare sage tea ^58^ or *Ginkgo biloba* extract ^59^).

Our statement also has been validated by the recent potential suggestion of the European Commission (according to press information ^7^) to consider hemp extracts as narcotic, and hence remove them from the possibility to be marketed as food or food supplement. As detailed elsewhere ^3, 41^, we believe that it would be disproportional to regulate CBD products as narcotic drug according to the principle of "ultima ratio" in criminal law. In closing, we would therefore like to note that we actually have suggested a regulated legalization of CBD products. Therefore, we question how or why the EIHA is interpreting this as the proposal of a “ban”.

## Conclusions

We hope our response informs the F1000Research readership about the most recent evidence regarding the toxicological and regulatory evaluation of CBD products. We believe that the Correspondence article of the EIHA ^1^ has made many unsubstantiated claims and is unable to discredit our scientific work that was based on a validated and externally accredited analytical method ^2^ with fully transparent criteria for risk assessment based on BfR ^19^ and EFSA ^26^.

We hope that the promised extensive toxicological studies and quality standards of EIHA will include the following research questions:

- The deviation of the content of commercial CBD preparations from the labelling consistently found in studies worldwide (see above) could partially derive from instability of CBD during storage ^60^. Research regarding stabilization of CBD appears necessary to ensure CBD stability during shelf-life.
- As a degradation of CBD is expected even in material from synthetic origin ^61^, the degradation products must be identified and toxicologically assessed.
- Avoidance of THC contamination and adherence to food standards for THC.
- Toxicological assessment of CBD as food ingredient aiming to identify acceptable daily intakes without risk for the consumer or pharmacological effects. Currently, there is no consensus of what constitutes a safe CBD dose, with recommendations ranging from as low as 4 mg/day ^62^ over 17.5 mg/day to 60 mg/day ^63^.
- Interactions between different compounds such as antagonistic or enhancing effects of the cannabinoid mixture contained in hemp extracts.

## Data availability

All data underlying the results are available as part of the article and no additional source data are required.
