# Supplementary material for: Evidence for adverse effects of cannabidiol (CBD) products and their non-conformity on the European food market – response to the European Industrial Hemp Association
Source: F1000Res. 2021 Aug 16;9:1051. Originally published 2020 Aug 26. [Version 2] doi: 10.12688/f1000research.26045.2 (PMC7542252; doi:10.12688/f1000research.26045.2)
Supplement: Peer review history

# Peer review: Reviewer response for version 1

**DOI:** 10.5256/f1000research.28744.r70380

**Published:** 2020-10-07

**Authors:** Katarina Cerne, Tomaž Pezdir

**Affiliations:** 1 Institute of Pharmacology and Experimental Toxicology, Faculty of Medicine, University of Ljubljana, Ljubljana, Slovenia; 2 Institute of Forensic Medicine, Faculty of Medicine, University of Ljubljana, Ljubljana, Slovenia

In this correspondence article, the authors, Lachenmeier *et al.* *,* attempt to clarify some points they made in their recent article entitled ''Are side effects of cannabidiol (CBD) products caused by tetrahydrocannabinol (THC) contamination?'' (version 3, 19 Aug 2020).  The reason for this response is comments on this article that were made by Kruse and Beitzke (26 Aug 2020) on behalf of the European Industrial Hemp Association (EIHA). The main topic is the safety of cannabidiol (CBD) products as a food or food ingredient for consumers, especially in relation to the tetrahydrocannabinol (THC) content. We believe that this issue is worth clarifying, because consumer exposure to unsafe substances is of particular concern since the exposed population may include people of all ages, both sexes and in all states of health. This area is of additional interest due to the new EU Novel Food Regulation 2015/2283.

 In order to understand the clarifications of Lachenmeier *et al*. (2020) in this correspondence, it is necessary first to read the comments of Kruse and Beitzke (2020) and the article of Lachenmeier *et al.* (2020).

 Below are the main points of Kruse and Beitzke comments to which Lachenmeier *et al.* wish to respond:

- Regarding the misunderstanding of the “THC” definition of daily dose of products,

Lachenmeier *et al*. explain that it should be clear from the method section and their definition of the abbreviation of THC as Δ ^9^-tetrahydrocannabinol that they only include psychotropic Δ ^9^-THC (“pure” Δ ^9^-THC) and not its precursor, non-psychotropic Δ ^9^-tetrahydrocannabinolic acid (THCA), in their risk assessment.

 From the description of the LC methods, it is obvious that the compounds were separated chromatographically. However, the mass spectrum does not show the difference (the parent masses of both TQS and QTOF are identical and, due to low fragmentation, the daughter ions are mainly the same on both instruments). Only the retention times from the chromatographic system show the difference between the substances. It would therefore be useful to show the chromatograms and note the retention times for each substance.

 EFSA also gives preference to assessing exposure to “pure” Δ ^9^-THC based on the LC-MS method. When samples are analysed using GC-FID or GC-MS (without a preliminary separation step) Δ ^9^-THC and Δ ^9^-THCA cannot be separated, so results are reported as the sum of Δ ^9^-THC/Δ ^9^-THCA. Additionally, EFSA considered 100 % conversion of THCA to THC. Such a report is thus expected to represent an overestimation of exposure to “pure” Δ ^9^-THC for two reasons: the THCA content in hemp is usually higher than the THC content and it is not known to what extent THCA is converted to THC in food ^1^.

 Kurse and Beitzke also comment on the very high daily dose of Δ9-THC in some products, e.g. tea products. Since this is an initial exposure assessment, it would be helpful if the authors were to describe major sources of uncertainties and the limitation of the exposure assessment in a separate paragraph.

  

- Side effects of CBD products

First, we would like to say that the term ‘side effect’ is inappropriate. Adverse or toxicological effect is a more appropriate term. However, we will use the term ‘side effect’ to avoid confusion. Since Kurse and Beitzke refuted any side effects of CBD, Lachenmeier *et al*. provided additional literature to the contrary. They proposed that the causes of the side effects are either a direct toxicological effect of CBD, the degradation of CBD to Δ ^9^-THC due to acidic hydrolysis in the stomach following oral consumption or Δ ^9^-THC directly contained in the products. A side effect such as “feeling high” is likely to be explained by Δ ^9^-THC contamination rather than by CBD. In humans, gastrointestinal effects are the most commonly reported side effect. Non-clinical studies have resulted in concerns about hepatotoxicity, teratogenicity and gut inflammation. They have also emphasized that the absence of acute toxic events does not mean that there are no chronic effects. However, not enough time has elapsed since CBD extracts have been more widely present on the market for chronic effect to show up (e.g., chronic liver injury). In addition, the absence of serious side effects is not enough for current safety criteria. Moreover, there is currently no system of nutri-vigilance implemented, so there is no formal registration of such cases and such events may be unrecognized.

 There are still many uncertainties and contradictions remaining from the increasing number of published cannabinoid safety studies. This is because these studies vary to the extreme in their methodology and quality, rendering results difficult to compare. Moreover, toxicity is not systematically covered, and there are no chronic toxicity data from well-defined exposure settings. Although Kurse and Beitzke criticize the usage of Epidiolex® as a reliable source of toxicological information on CBD, as a 99 % pure extract from *C. sativa*, CBD has become the most extensively toxicologically tested cannabinoid. In spite of the fact that Epidiolex® has been approved for treatment-resistant epilepsy, the non-clinical part of testing was independent of this indication ^2^. CBD is extensively metabolised in the liver and gut, mainly by the CYP2C19, CYP3A4, UGT1A7, UGT1A9, and UGT2B7 enzymes. The metabolism of CBD is very complex, especially in hepatocytes. The main human metabolite is 7-carboxy-cannabidiol (7-COOH-CBD; ~90 % of all drug-related substances measured in the plasma) ^3^. Its toxicological profile has not been investigated because experimental animals for toxicological studies (mice, rats, and dogs) do not metabolise CBD to a comparable extent as do humans ^4^. The major concern with 7-COOH-CBD may be its reactive acyl-glucuronide ^5^. CYP-mediated interaction is one of the major concerns in clinical practice. Drug interaction trials to assess the effect of CBD on these enzymes in healthy volunteers will therefore be conducted during the post-marketing period. A full battery of non-clinical oral reproductive and developmental studies has been conducted with purified CBD, showing toxicological effects ^2^. Since the novel food status of CBD extracts was confirmed in January 2019, the Food Standard Agency (FSA) has given CBD companies a deadline of March 31, 2020 to apply for authorisations of their CBD extracts and isolates (for each product). In this regard, toxicity testing should be systematically covered, so the safety of each of CBD product will be clear.

  

- Mitigation of THC effects by interaction with CBD

Lachenmeier *et al*. re-confirmed their risk assessment without considering the interaction with CBD, since the opinion of EFSA is that information on THC/CBD interactions is controversial and not consistently antagonistic. EFSA also re-confirmed their acute reference dose (ARfD) of 1 µg/kg body weight (BW), without considering the interaction with CBD. Additionally, Lachenmeier *et al*. provided reference to recent research of Solowij *et al.,* in which they found that the effects of Δ ^9^-THC may even be enhanced by low-dose CBD.

 In spite of its low affinity for CB _1_ and CB _2 _receptors, CBD can interfere with some THC adverse effects, particularly in the brain, without interfering with the intended THC effects, such as muscle relaxation ^6^. In terms of the pharmacokinetic CBD/THC interaction, CBD may impair THC hydrolysis by the CYP450 enzyme ^7^. The inhibition of THC metabolism may vary with species, timing of administration (CBD pre-administration *vs* co-administration), and CYP isoenzymes ^8^. Whether CBD will antagonise or potentiate THC effects also seems to depend on their administration ratio, and this ratio varies with species. Given all these possibilities, prediction of interactions between THC and CBD in food can be quite a challenge, so we do not yet see the possibility of including them in the risk assessment of CBD products.

 When using ARfD, it is necessary to consider that it represents an estimate of the amount of a substance in food that can be ingested, usually during one meal or one day. This has been established on the base of central nervous system effects and increased heart rate, which were noted at a low Δ ^9^-THC level (2.5 mg/day) in humans and occurred within a short time after dose administration.  This dose, corresponding to 0.036 mg/day/kg BW for a person with a body weight of 70 kg, was regarded as the lowest-observed-adverse-effect level (LOAEL) in both single and repeated studies. Using an uncertainty factor (UF) of 3 for extrapolation from the LOAEL to a no-observed-effect-level (NOAEL) and an UF of 10 for interindividual differences, an overall UF of 30 was established (0.036 mg/day/kg: 30 = 0.001 mg/day/kg) (1). When applying the UF, the recommended daily dose for adults is 0.07 mg.  At a higher consumption level, there is a risk of influence on the person’s ability to operate machinery and drive vehicles. Interestingly, Lachenmeier *et al*. used LOAEL in their assessment without applying UF. 

- Illegality of all hemp products containing isolated CBD or hemp extracts

After the date mentioned above, only products that have a validated novel food application will be allowed to remain on the market. It is therefore our view that regulatory issues are very extensively written and could be less extensive and shorter.

Are arguments sufficiently supported by evidence from the published literature or by new data and results?

Partly

Is the conclusion balanced and justified on the basis of the presented arguments?

Yes

Is the rationale for commenting on the previous publication clearly described?

Yes

Are any opinions stated well-argued, clear and cogent?

Yes

Reviewer Expertise:

Katarina Černe: experimental and clinical toxicology, pharmacology, cannabinoids, risk assessment. Tomaž Pezdir: analytical chemistry, forensic toxicology, cannabinoids

We confirm that we have read this submission and believe that we have an appropriate level of expertise to confirm that it is of an acceptable scientific standard.

## References

[1] : Acute human exposure assessment to tetrahydrocannabinol (Δ9-THC).*EFSA J*.2020;18(1) : 10.2903/j.efsa.2020.5953e0595310.2903/j.efsa.2020.595332626501PMC7008849

[2] International non-proprietary name: cannabidiol. *European Medicines Agency*.2019; Reference source

[3] : Human Metabolites of Cannabidiol: A Review on Their Formation, Biological Activity, and Relevance in Therapy.*Cannabis Cannabinoid Res*.2016;1(1) : 10.1089/can.2015.001290-10110.1089/can.2015.001228861484PMC5576600

[4] : Comparative metabolism of cannabidiol in dog, rat and man. *Pharmacology Biochemistry and Behavior*.1991;40(3) : 10.1016/0091-3057(91)90358-9523-53210.1016/0091-3057(91)90358-91806942

[5] : Acyl glucuronides: the good, the bad and the ugly.*Biopharm Drug Dispos*.2010;31(7) : 10.1002/bdd.720367-9510.1002/bdd.72020830700

[6] : A tale of two cannabinoids: the therapeutic rationale for combining tetrahydrocannabinol and cannabidiol.*Med Hypotheses*.2006;66(2) : 10.1016/j.mehy.2005.08.026234-4610.1016/j.mehy.2005.08.02616209908

[7] : Effect of cannabidiol pretreatment on the kinetics of tetrahydrocannabinol metabolites in mouse brain.*Drug Metab Dispos*.1995;23(8) :825-317493549

[8] : Towards a better cannabis drug.*Br J Pharmacol*.2013;170(7) : 10.1111/bph.124001363-410.1111/bph.1240024024867PMC3838682

# Response

**Published:** 2021-08-11

**Authors:** Dirk W. Lachenmeier

**Affiliations:** Chemisches und Veterinäruntersuchungsamt (CVUA) Karlsruhe, Germany

We thank the reviewers for the assessment of our article. Due to the requests, the following changes were implemented:

- A representative chromatogram with marked retention times for each substance was included.
- A short discussion about sources of uncertainties and limitations of exposure assessment was added to the section “THC definition and estimation of daily dose of products”.
- The term “side effects” was changed to “adverse effects” as requested.
- Regarding the adverse effects of CBD products, we want to thank the reviewer for the insights and remarks on toxicology and metabolism of the compound. We found that this information was more appropriate for inclusion in our original article and have done so during our recent revision into v4 of the article ^1^.
- Regarding the application of ARfD and LOAEL for risk assessment, we want to point out that were are using both thresholds and not only the LOAEL. A food containing THC in exceedance of ARfD is considered as being “unfit for human consumption” (Article 14 No. 2 (b) of regulation (EC) No 178/2002), while a food containing THC in exceedance of LOAEL is considered as “injurious to health” (Article 14 No. 2 (a) of regulation (EC) No 178/2002). The distinction is also clearly marked by the colours in Table 2 (last column) of our original article ^1^. Furthermore, this distinction also leads to prioritization of risk management measures, e.g. in terms of urgency, inclusion in rapid alert system RASFF, etc.
- In conclusion, it must be noted that while having already passed the mentiond FSA deadline of March 31, 2021, no product has been approved so far in the UK, and none in the European Union as well. Therefore, we believe that our regulatory section is still valid, and we have refrained from shortening the section so far.

**References**

**1 **Lachenmeier DW, Habel S, Fischer B et al. Are side effects of cannabidiol (CBD) products caused by tetrahydrocannabinol (THC) contamination? [version 4; peer review: 2 approved, 1 approved with reservations]. F1000Research 2021, 8:1394 ( https://doi.org/10.12688/f1000research.19931.4)

# Peer review: Reviewer response for version 1

**DOI:** 10.5256/f1000research.28744.r70375

**Published:** 2020-09-07

**Authors:** Patrick John O'Mahony

**Affiliations:** 1 Food Safety Authority of Ireland, Dublin, Ireland

**Side effects of CBD products - **EU food regulators await the final opinion of EFSA on the safety assessment of synthetic CBD. Many claims are made about CBD, some health claims not authorised in the EU, some relating to the mitigating effect of CBD on THC (as yet unproven conclusively). World-wide reports of foods and supplements claiming to contain CBD show that a significant proportion also contain THC, some at unsafe levels and some at levels that could result in the "high" associated with recreational cannabis use.

**THC definition and estimation of daily dose of products - **In some EU Member States (MS) there is a threshold for THC contaminants in food, but not in others. EU food law is categorical (Reg 178/2002) food does not include narcotics which means any THC in food is considered a contaminant for which there is as yet no safe limit designated. Some EU MS have developed national limits for THC in food and in the future there could be an EU threshold for THC as a contaminant in hemp seed food products (in development). In some MS police can and do remove food products from sale based on any THC level under national legislation.

**Cannabis shot  - **I agree with the author here. Under food law, a supplement must declare a recommended and/or maximum dosage. A shot is generally considered a small amount of concentrated beverage (usually alcoholic) to be consumed in one go.

**CBD oil - **Supplements must declare a dosage by law. If not a supplement with no recommended usage guidance then it is anyone's guess as to appropriate use.

**Mitigation of THC effects by interaction with CBD - **This is not an argument for food law and has not been discussed by regulators. THC presence in food is not specifically permitted by food law and tolerance thresholds for THC as a contaminant are in effect in some EU MS only, with a possibility of an EU-wide threshold in the future. It is unlikely that any EU food regulatory authority would have such a discussion with the industry.

**Illegality of all hemp products containing isolated CBD or hemp extracts - **The novel food catalogue is not a legally binding document as stated. However, it does reflect the agreed outcome of discussions at the novel food working group and therefore is binding in so far as all MS agree to interpret and implement the conclusions accordingly within their jurisdictions. Industry groups have tried unsuccessfully to argue that hemp extracts were on the EU market prior to 1997. Any food or ingredient which is a purified/extracted/concentrated component of an existing (not novel) food is likely to be considered a novel food and this standard has been used for almost two decades with many different examples available. Court rulings in one MS have no bearing on the implementation or interpretation of food law in other MS. At most such national court rulings can result in a case being sent to the European Court of Justice (ECJ), the results of which may then necessitate EU-wide legislation consideration.

 Novel food status is not based on safety or otherwise as stated by the author, but on a significant history of consumption in the EU before 1997.

**Judgement about food producers of CBD products - **The idea that registered CBD producers of hemp extracts would be allowed on the market was dismissed as an irrational proposal of industry regulation by this regulatory body. THC content is not the only regulatory or safety issue with hemp products on the market as pointed out by the author through the many reports cited.

**Judgement of the hemp industry in the food sector - **The statement in Newsweek about "the largest uncontrolled clinical trials in history" is in my opinion the type of histrionic argument used against GMOs and in other food and non-food areas over the years. The reality is that many foods on the market (particularly supplements) fly under the radar until caught by routine surveillance or by the manifestation of safety issues. Therefore, it could be said that there are many "large-scale uncontrolled clinical trials" ongoing around the world in food, cosmetics, etc. This sort of inflammatory statement does not aid a rational discussion. There has been no reliable safety assessment yet of CBD ingestion and until then, safety levels of CBD or the hundred plus other cannabinoids in *Cannabis sativa* is unclear. A thorough safety assessment as being carried out by EFSA currently will address issues of safe intake levels, interactions of CBD with other cannabinoids or other foods. It will also address stability and breakdown products of cannabinoids like CBD.

Are arguments sufficiently supported by evidence from the published literature or by new data and results?

Yes

Is the conclusion balanced and justified on the basis of the presented arguments?

Yes

Is the rationale for commenting on the previous publication clearly described?

Yes

Are any opinions stated well-argued, clear and cogent?

Yes

Reviewer Expertise:

I am not a research scientist. I am a scientist (molecular biologist by training) working in the Irish/EU food regulatory arena for 20 years. I have been looking after the novel food area (among others) in Ireland for 20 years and have coordinated the analytical survey of the Irish market for hemp-products as published in February 2020.

I confirm that I have read this submission and believe that I have an appropriate level of expertise to confirm that it is of an acceptable scientific standard.

# Response

**Published:** 2021-08-11

**Authors:** Dirk W. Lachenmeier

**Affiliations:** Chemisches und Veterinäruntersuchungsamt (CVUA) Karlsruhe, Germany

Thank you for your insightful remarks regarding the regulatory status of CBD. As requested, we have removed the histrionic argument from Newsweek in v4 of our article ^1^.

**References**

**1 **Lachenmeier DW, Habel S, Fischer B et al. Are side effects of cannabidiol (CBD) products caused by tetrahydrocannabinol (THC) contamination? [version 4; peer review: 2 approved, 1 approved with reservations]. F1000Research 2021, 8:1394 ( https://doi.org/10.12688/f1000research.19931.4)
